# Spin-structures of the Bose-Einstein condensates with three kinds of spin-1 atoms

**DOI:** 10.1038/s41598-020-59540-z

**Published:** 2020-02-17

**Authors:** Y. Z. He, Y. M. Liu, C. G. Bao

**Affiliations:** 1 School of Physics, Sun Yat-Sen University, Guangzhou, 510275 P.R. China; 2 Department of physics, Shaoguan University, Shaoguan, 510205 P.R. China

**Keywords:** Applied mathematics, Bose-Einstein condensates

## Abstract

We have performed a quantum mechanic calculation (including solving the coupled Gross-Pitaevskii equations to obtain the spatial wave functions, and diagonalizing the spin-dependent Hamiltonian in the spin-space to obtain the total spin state) together with an analytical analysis based on a classical model. Then, according to the relative orientations of the spins *S*_*A*_, *S*_*B*_ and *S*_*C*_ of the three species, the spin-structures of the ground state can be classified into two types. In Type-I the three spins are either parallel or anti-parallel to each other, while in Type-II they point to different directions but remain to be coplanar. Moreover, according to the magnitudes of *S*_*A*_, *S*_*B*_ and *S*_*C*_, the spin-structures can be further classified into four kinds, namely, *p* + *p* + *p* (all atoms of each species are in singlet-pairs), one species in f (fully polarized) and two species in *q* (a mixture of polarized atoms and singlet-pairs), two in *f* and one in *q*, and *f* + *f* + *f*. Other combinations are not allowed. The scopes of the parameters that supports a specific spin-structure have been specified. A number of spin-structure-transitions have been found. For Type-I, the critical values at which a transition takes place are given by simple analytical formulae, therefore these values can be predict.

## Introduction

The study of the multi-species Bose-Einstein condensates (BEC) with atoms having nonzero spin is an attractive topic^1^. For these systems, when the temperature is extremely low (say, lower than 10^−9^ K), the spatial degrees of freedom are nearly frozen and the spin degrees of freedom play essential roles^2,3^. Various spin-structures will emerge, and they are found to be sensitive to the very weak spin-dependent forces. Therefore, these systems might be ideal for realizing exquisite control.

When the BEC contains only one kind of *N* spin-1 atoms, the polar phase (*p*-phase) and the ferromagnetic phase (*f*-phase) have been found in the ground state (g.s.)^4–10^. In the *p*-phase, the spins of atom are two-by-two coupled to zero to form the singlet pairs (*s*-pair), and the total spin of the condensate *S* = 0. In the *f*-phase, all the spins are fully polarized, i.e., lying along a common direction, and *S* = *N*. For 2-species BEC, it was found in^1,11–23^ that there are three types of spin-structures, namely, (i) the *p* + *p* spin-structure where both species are in *p*-phase; (ii) the *f*//*f* structure where both species are in *f*-phase, and the two total spins (each for a species) are lying either parallel or antiparallel to each other; and (iii) the *f*//*q* structure where one in *f*-phase and one in quasi-ferromagnetic phase (*q*-phase, a mixture of aligned spins and *s*-pairs).

The above message from 2-species BEC attracts the exploration on the spin-structures of multi-species BEC. Note that, for 3-species BEC, the three intra-species and three inter-species spin-dependent interactions can be repulsive or attractive. Thus, the spin-structures are expected to be very rich. However, this interesting topic is scarcely studied before. This paper is a primary study on this topic. The aim is to clarify the variety of the spin-structures and the related critical phenomena, and the effects of the intra- and inter-species interactions. We believe that the knowledge extracted from 3-species BEC would be in general useful for understanding the spin-structures of many-body systems with multi-species.

We proceed in the following way:

- From the experience of 2-species BEC, the spin-structures are seriously affected by the compactness of the spatial wave functions (i.e., $\int \,{\varphi }_{A}^{4}{\rm{d}}{\bf{r}}$ and $\int \,{\varphi }_{B}^{4}{\rm{d}}{\bf{r}}$) and the overlap (i.e., $\int \,{\varphi }_{A}^{2}{\varphi }_{B}^{2}{\rm{d}}{\bf{r}}$). For 3-species BEC, $\int \,{\varphi }_{J}^{4}{\rm{d}}{\bf{r}}$ (*J* = *A*, *B*, *C*) and $\int \,{\varphi }_{J}^{2}{\varphi }_{J{\prime} }^{2}{\rm{d}}{\bf{r}}$ are believed to be also important. Therefore, we solve the coupled Gross-Pitaevskii equations (CGP) under the Thomas-Fermi approximation (TFA, in which the kinetic energies have been neglected) to obtain the spatial wave functions. Since the kinetic energy increases linearly with particle number *N*, while the interaction energy increases with *N*^2^, the relative importance of the kinetic terms is very weak when *N* is very large. Therefore, the TFA is applicable when the particle numbers are huge as usually in the experiments of BEC (numerical estimations are referred to^24–26^).
- Let *S*_*J*_ be the total spin of the *J*-species and *S* be the total spin of the mixture. Let Ξ denote the total spin-state of the mixture. When the singlet-pairing force has been neglected, Ξ has the three {*S*_*J*_} and *S* as good quantum numbers. Ξ can be obtained via a diagonalization of the Hamiltonian in the spin-space. In order to extract physical features from Ξ, in addition to the good quantum numbers, the averaged angles ${\bar{\theta }}_{JJ{\prime} }$ between *S*_*J*_ and ${S}_{J{\prime} }$ have also been calculated. Thereby various types of spin-structures specified by {*S*_*J*_} and $\{{\bar{\theta }}_{JJ{\prime} }\}$ can be identified, and the transitions among them are found.
- In addition to the above quantum mechanic (QM) calculation, a corresponding classical model has been proposed and solved analytically. The results from the model are checked via a comparison with those from QM calculation. This model helps greatly to understand the complicated 3-species spin-structures.

## Hamiltonian and the Ground State

Let the mixture of three kinds of spin-1 atoms be trapped by isotropic and harmonic species-dependent potentials $\frac{1}{2}{m}_{J}{\omega }_{J}^{2}{r}^{2}$. The intra-species interaction is ${V}_{J}={\sum }_{1\le i < j\le {N}_{J}}\,\delta ({{\bf{r}}}_{i}-{{\bf{r}}}_{j})({c}_{J0}+{c}_{J2}{{\bf{F}}}_{i}^{J}\cdot {{\bf{F}}}_{j}^{J})$, where ${{\bf{F}}}_{i}^{J}$ is the spin operator of the *i*-th atom of the *J*-species. The inter-species interaction is ${V}_{JJ{\prime} }={\sum }_{1\le i\le {N}_{J}}\,{\sum }_{1\le j\le {N}_{J{\prime} }}$ $\delta ({{\bf{r}}}_{i}-{{\bf{r}}}_{j})({c}_{JJ{\prime} 0}+{c}_{JJ{\prime} 2}{{\bf{F}}}_{i}^{J}\cdot {{\bf{F}}}_{j}^{J{\prime} })$. We introduce two quantities *m* and *ω*, and use $\hslash \omega$ and $\lambda \equiv \sqrt{\hslash /(m\omega )}$ as the units for energy and length. Then, the total Hamiltonian is

1 $$H=\sum _{J}\,({\hat{K}}_{J}+{V}_{J})+\sum _{J < J{\prime} }\,{V}_{JJ{\prime} },$$

where $${\hat{K}}_{J}={\sum }_{i\mathrm{=1}}^{{N}_{J}}\,{\hat{h}}_{J}(i)$$, $${\hat{h}}_{J}(i)=\frac{1}{2}(-\frac{m}{{m}_{J}}{\nabla }_{i}^{2}+{\gamma }_{J}{r}_{i}^{2})$$ and $${\gamma }_{J}=\frac{{m}_{J}{\omega }_{J}^{2}}{m{\omega }^{2}}$$.

Note that, in the ground state (g.s.), every particles of a kind will condense to a spatial state (say, ${\varphi }_{J}$) which is most favorable for binding. Let Ξ denotes a normalized total spin-state. Then the g.s. can be in general written as

2 $${\Psi }_{{\rm{o}}}=\mathop{\prod }\limits_{i\mathrm{=1}}^{{N}_{A}}\,{\varphi }_{A}({{\bf{r}}}_{i})\mathop{\prod }\limits_{j\mathrm{=1}}^{{N}_{B}}\,{\varphi }_{B}({{\bf{r}}}_{j})\mathop{\prod }\limits_{k\mathrm{=1}}^{{N}_{C}}\,{\varphi }_{C}({{\bf{r}}}_{k})\,\Xi .$$

Let ${\vartheta }_{{S}_{J}{M}_{J}}^{{N}_{J}}$ denote a normalized and all-symmetric spin-state for the *J*-species where the spins are coupled to ${S}_{J}$ and its *Z*-component *M*_*J*_. According to the theory given in^27^, ${N}_{J}-{S}_{J}$ must be even, the multiplicity of ${\vartheta }_{{S}_{J}{M}_{J}}^{{N}_{J}}$ is one (i.e., ${\vartheta }_{{S}_{J}{M}_{J}}^{{N}_{J}}$ is unique when *S*_*J*_ and *M*_*J*_ are fixed), and the set $\{{\vartheta }_{{S}_{J}{M}_{J}}^{{N}_{J}}\}$ is complete for all-symmetric spin-states. Let ${({\vartheta }_{{S}_{A}}^{{N}_{A}}{\vartheta }_{{S}_{B}}^{{N}_{B}})}_{{S}_{AB}{M}_{AB}}\equiv {({S}_{A}{S}_{B})}_{{S}_{AB}{M}_{AB}}$ be a combined spin-state of the *A*- and *B*-species, in which *S*_*A*_ and *S*_*B*_ are coupled to ${S}_{AB}$ and ${M}_{AB}$. Let ${(({\vartheta }_{{S}_{A}}^{{N}_{A}}{\vartheta }_{{S}_{B}}^{{N}_{B}}{)}_{{S}_{AB}}{\vartheta }_{{S}_{C}}^{{N}_{C}})}_{SM}\equiv {(({S}_{A}{S}_{B}{)}_{{S}_{AB}}{S}_{C})}_{SM}$ be a total spin-state of the mixture, in which ${S}_{AB}$ and ${S}_{C}$ are further coupled to $S$ and $M$. It is recalled that ${S}_{A}$, ${S}_{B}$, ${S}_{C}$, $S$ and $M$ are good quantum numbers, but ${S}_{AB}$ is not. Nonetheless, the states ${(({S}_{A}{S}_{B}{)}_{{S}_{AB}}{S}_{C})}_{SM}$ form a complete set so that Ξ can be expanded by them.

### The coupled gross-pitaevskii equations and the spatial wave functions

For the Hamiltonian given in Eq. (1), the associated CGP equations for ${\varphi }_{A}$ to ${\varphi }_{C}$ are^11,28^

3 $$({\hat{h}}_{A}+{\alpha }_{AA}{\varphi }_{A}^{2}+{\alpha }_{AB}{\varphi }_{B}^{2}+{\alpha }_{CA}{\varphi }_{C}^{2}-{\varepsilon }_{A}){\varphi }_{A}=0$$

4 $$({\hat{h}}_{B}+{\alpha }_{AB}{\varphi }_{A}^{2}+{\alpha }_{BB}{\varphi }_{B}^{2}+{\alpha }_{BC}{\varphi }_{C}^{2}-{\varepsilon }_{B}){\varphi }_{B}=0$$

5 $$({\hat{h}}_{C}+{\alpha }_{CA}{\varphi }_{A}^{2}+{\alpha }_{BC}{\varphi }_{B}^{2}+{\alpha }_{CC}{\varphi }_{C}^{2}-{\varepsilon }_{C}){\varphi }_{C}=0$$

where ${\varphi }_{A}$, ${\varphi }_{B}$ and ${\varphi }_{C}$ are required to be normalized.

Since the spin-dependent forces are in general two order weaker than the spin-independent forces (say, $|{c}_{J2}/{c}_{J0}|=0.0047$ for ^87^Rb, and 0.0313 for ^23^Na), as a reasonable approximation, the contribution of the former can be neglected. Then, we have ${\alpha }_{JJ{\prime} }\simeq {c}_{J0}{N}_{J}$ (if $J=J{\prime}$) or ${\alpha }_{JJ{\prime} }={c}_{JJ{\prime} 0}{N}_{J{\prime} }$ (if $J\ne J{\prime}$).

Under the TFA where the terms of kinetic energy have been neglected, in a domain where all the ${\varphi }_{J}$ are nonzero, the CGP can be written in a matrix form as

6 $${\mathfrak{M}}(\begin{array}{c}{\varphi }_{A}^{2}\\ {\varphi }_{B}^{2}\\ {\varphi }_{C}^{2}\end{array})=(\begin{array}{c}{\varepsilon }_{A}-{\gamma }_{A}{r}^{2}\mathrm{/2}\\ {\varepsilon }_{B}-{\gamma }_{B}{r}^{2}\mathrm{/2}\\ {\varepsilon }_{C}-{\gamma }_{C}{r}^{2}\mathrm{/2}\end{array}),$$

where ${\mathfrak{M}}$ is a 3 × 3 matrix with elements ${\alpha }_{JJ{\prime} }$. Let the determinant of ${\mathfrak{M}}$ be ${\mathfrak{D}}$. From the above matrix equation, we obtain a formal solution of the CGP as

7 $${\varphi }_{J}^{2}={Z}_{J}-{Y}_{J}{r}^{2},\,(J=A,B,C)$$

8 $${Z}_{J}={{\mathfrak{D}}}_{J}^{Z}/{\mathfrak{D}}.$$

${{\mathfrak{D}}}_{J}^{Z}$ is a determinant obtained by changing the *J* column of ${\mathfrak{D}}$ from $({\alpha }_{AJ},{\alpha }_{BJ},{\alpha }_{CJ})$ to $({\varepsilon }_{A},{\varepsilon }_{B},{\varepsilon }_{C})$.

9 $${Y}_{J}={{\mathfrak{D}}}_{J}^{Y}/{\mathfrak{D}}.$$

${{\mathfrak{D}}}_{J}^{Y}$ is also a determinant obtained by changing the *J* column of ${\mathfrak{D}}$ to $({\gamma }_{A}\mathrm{/2,}{\gamma }_{B}\mathrm{/2,}{\gamma }_{C}\mathrm{/2)}$. Once all the parameters are given, the three ${Y}_{J}$ are known because they depend only on ${\alpha }_{JJ{\prime} }$ and ${\gamma }_{J}$. However, the three ${Z}_{J}$ have not yet been known because they depend on $({\varepsilon }_{A},{\varepsilon }_{B},{\varepsilon }_{C})$. When ${Y}_{J}$ is positive (negative), ${\varphi }_{J}^{2}$ goes down (up) with *r*. Thus, the main feature of this formal solution depends on the signs of the set $\{{Y}_{J}\}$.

The set $\{{Z}_{J}\}$ and the set $\{{\varepsilon }_{J}\}$ are related as

10 $${\varepsilon }_{J}=\sum _{J{\prime} }\,{\alpha }_{JJ{\prime} }{Z}_{J{\prime} },$$

11 $${Z}_{J}=\sum _{J{\prime} }\,{\bar{\alpha }}_{JJ{\prime} }{\varepsilon }_{J{\prime} }.$$

where ${\bar{\alpha }}_{JJ{\prime} }={{\mathfrak{d}}}_{J{\prime} J}/{\mathfrak{D}}$, and ${{\mathfrak{d}}}_{J{\prime} J}$ is the algebraic cominor of ${\alpha }_{J{\prime} J}$. This formal solution is named the Form III, which is valid only in a domain where all the three ${\varphi }_{J}$ are nonzero.

When two wave functions are nonzero inside a domain while the third is zero, in a similar way we obtain

12 $$\{\begin{array}{l}{\varphi }_{l}^{2}={Z}_{l}^{(n)}-{Y}_{l}^{(n)}{r}^{2}\\ {\varphi }_{m}^{2}={Z}_{m}^{(n)}-{Y}_{m}^{(n)}{r}^{2}\\ {\varphi }_{n}^{2}=0\end{array},$$

where *l*, *m* and *n* are a cyclic permutation of *A*, *B* and *C*.

13 $$\{\begin{array}{l}{Z}_{l}^{(n)}=({\alpha }_{mm}{\varepsilon }_{l}-{\alpha }_{lm}{\varepsilon }_{m})/{{\mathfrak{d}}}_{nn}\\ {Y}_{l}^{(n)}=\frac{1}{2}({\alpha }_{mm}-{\alpha }_{lm})/{{\mathfrak{d}}}_{nn}\\ {Z}_{m}^{(n)}=({\alpha }_{ll}{\varepsilon }_{m}-{\alpha }_{ml}{\varepsilon }_{l})/{{\mathfrak{d}}}_{nn}\\ {Y}_{m}^{(n)}=\frac{1}{2}({\alpha }_{ll}-{\alpha }_{ml})/{{\mathfrak{d}}}_{nn}\end{array}$$

Once the parameters are given, the six ${Y}_{n{\prime} }^{(n)}$ ($n{\prime} \ne n$) are known, while the six ${Z}_{n{\prime} }^{(n)}$ have not yet. This formal solution with ${\varphi }_{n}=0$ is denoted as Form II_*n*_, where the subscript specifies the vanishing wave function.

When one and only one of the wave functions is nonzero in a domain (say, ${\varphi }_{J}\ne 0$), it must have the unique form as

14 $${\varphi }_{J}^{2}=\frac{1}{{\alpha }_{JJ}}({\varepsilon }_{J}-{\gamma }_{J}{r}^{2}\mathrm{/2}).$$

Obviously, ${\varphi }_{J}$ in this form must descend with $r$. This form is denoted as Form I_*J*_, where the subscript specifies the survived wave function.

If a wave function (say, ${\varphi }_{J}$) is nonzero in a domain but becomes zero when $r\ge {r}_{{\rm{o}}}$, then a downward form-transition (say, from Form III to II_*j*_) will occur at ${r}_{{\rm{o}}}$. Whereas if ${\varphi }_{J}$ is zero in a domain but emerges from zero when $r\ge {r}_{{\rm{o}}}$, then an upward form-transition (say, from Form II_*J*_ to III) will occur at ${r}_{{\rm{o}}}$. ${r}_{{\rm{o}}}$ appears as the boundary separating the two connected domains, each supports a specific form. In this way, the formal solutions serve as the building blocks, and they will link up continuously to form an entire solution of the CGP. They must be continuous at the boundary because the two sets of wave functions by the two sides of the boundary satisfy exactly the same set of nonlinear equations at the boundary.

Recall that there are three unknowns ${\varepsilon }_{A}$, ${\varepsilon }_{B}$ and ${\varepsilon }_{C}$ contained in the formal solutions. Taking the three additional equations of normalization $\int {\varphi }_{J}^{2}{\rm{d}}{\bf{r}}=1$ into account, the three unknowns can be obtained. Then, under the TFA, the CGP is completely solved. The details are shown below.

### The spatial wave functions

**Figure 1:**
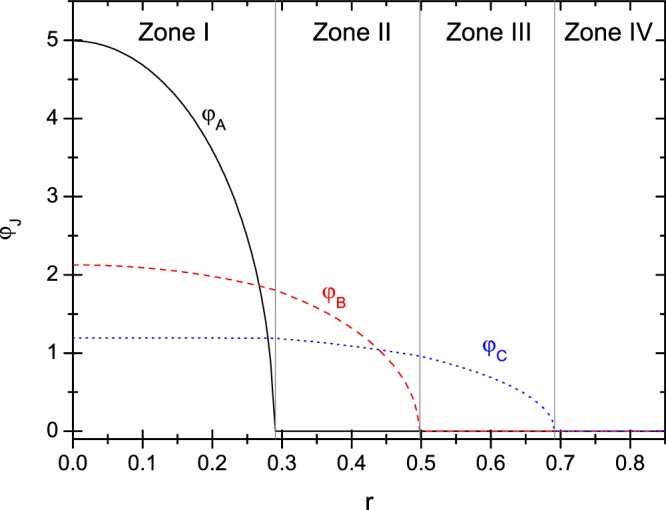
An example of the spatial wave functions of a miscible state obtained from the TFA solution of the CGP. The parameters are given as ${Y}_{A}=300$, ${Y}_{B}=15$, ${Y}_{C}=0.1$, ${Y}_{B}^{(A)}=20$, ${Y}_{C}^{(A)}=3$, ${\alpha }_{CC}=0.01$ and ${\gamma }_{C}=0.08$.

The spin-structures in multi-species BEC is caused by the inter-species interactions. Obviously, they would act more effectively when the three species are distributed closer to each other. Therefore, in the following examples, we take the miscible states into account, in which all the three species have nonzero distribution at the center (*r* = 0). An example is given in Fig. 1, where the wave functions in zone I to IV are in Form III, Form II_1_, Form I_3_, and empty, respectively.

For this example, we know that the boundary ${r}_{a}$ (at which ${\varphi }_{A}=0$) is equal to $\sqrt{{Z}_{A}/{Y}_{A}}$ (refer to Eq. (7)), ${r}_{b}$ (at which ${\varphi }_{B}=0$) is equal to $\sqrt{{Z}_{B}^{(A)}/{Y}_{B}^{(A)}}$ (Eq. (12)), ${r}_{c}$ (at which ${\varphi }_{C}=0$) is equal to $\sqrt{2{\varepsilon }_{C}/{\gamma }_{C}}$ (Eq. (14)). They give the outmost boundary of ${\varphi }_{A}$, ${\varphi }_{B}$ and ${\varphi }_{C}$, respectively. Taking the normalization into account, we obtain

15 $${Z}_{A}={(\frac{15}{8\pi })}^{\mathrm{2/5}}{Y}_{A}^{\mathrm{3/5}},$$

16 $${Z}_{B}^{(A)}={(\frac{15}{8\pi })}^{\mathrm{2/5}}{({Y}_{B}^{(A)})}^{\mathrm{3/5}}{\mathrm{[1}-({Y}_{B}-{Y}_{B}^{(A)})/{Y}_{A}]}^{\mathrm{2/5}},$$

17 $${Z}_{B}={Z}_{B}^{(A)}+{(\frac{15}{8\pi })}^{\mathrm{2/5}}({Y}_{B}-{Y}_{B}^{(A)})/{Y}_{A}^{\mathrm{2/5}},$$

18 $${\varepsilon }_{C}/{\alpha }_{CC}={(\frac{15}{8\pi })}^{\mathrm{2/5}}{(\frac{{\gamma }_{C}}{2{\alpha }_{CC}})}^{\mathrm{3/5}}{[1-\frac{{Y}_{C}-{Y}_{C}^{(A)}}{{Y}_{A}}-({Y}_{C}^{(A)}-\frac{{\gamma }_{C}}{2{\alpha }_{CC}})\frac{1}{{Y}_{B}^{(A)}}(1-\frac{{Y}_{B}-{Y}_{B}^{(A)}}{{Y}_{A}})]}^{\mathrm{2/5}},$$

19 $${Z}_{C}^{(A)}=\frac{{\varepsilon }_{C}}{{\alpha }_{CC}}+({Y}_{C}^{(A)}-\frac{{\gamma }_{C}}{2{\alpha }_{CC}})\frac{{Z}_{B}^{(A)}}{{Y}_{B}^{(A)}},$$

20 $${Z}_{C}={Z}_{C}^{(A)}+({Y}_{C}-{Y}_{C}^{(A)}){(\frac{15}{8\pi {Y}_{A}})}^{\mathrm{2/5}}.$$

Since ${Z}_{A}$, ${Z}_{B}$, and ${Z}_{C}$ have been obtained as given above, ${\varepsilon }_{A}$ and ${\varepsilon }_{B}$ can be further obtained via Eq. (10). Then, the entire solution of the CGP together with the chemical potentials are completely known.

Nonetheless, the realization of the miscible state is based on a number of assumptions. First, it is assumed that all the wave functions are nonzero at the center, thus ${Z}_{A} > 0$, ${Z}_{B} > 0$, and ${Z}_{C} > 0$ are required. Second, ${\varphi }_{A}$ is assumed to descend with $r$ in zone I and ${\varphi }_{B}$ is assumed to descend with $r$ in zone II, thus ${Y}_{A} > 0$ and ${Y}_{B}^{(A)} > 0$ are required. Third, ${\varphi }_{B}{|}_{{r}_{a}} > 0$ and ${\varphi }_{C}{|}_{{r}_{a}} > 0$ are required so that the Form III can link with a Form II_*A*_ at ${r}_{a}$. Fourth, ${\varphi }_{C}{|}_{{r}_{b}} > 0$ is required so that the Form II_*A*_ can link with a Form I_*C*_ at ${r}_{b}$. Each of these requirements will impose a constraint on the parameters (say, the requirement ${\varphi }_{B}{|}_{{r}_{a}} > 0$ leads to ${Z}_{B}^{(A)} > {Y}_{B}^{(A)}{r}_{a}^{2}$, and therefore leads to ${Y}_{A} > {Y}_{B}$). Thus, the type as shown in Fig. 1 can be realized only if the parameters are given inside a specific scope. A comprehensive discussion on the scope of parameters for each spatial type of solution is the base for obtaining the phase-diagrams, but this is beyond the scope of this paper.

### The total spin-state

Making use of the spin basis-state, we define a set of basis-states for the g.s. as

21 $${\psi }_{{\mathfrak{S}},{S}_{AB}}=\mathop{\prod }\limits_{i\mathrm{=1}}^{{N}_{A}}\,{\phi }_{A}({{\bf{r}}}_{i})\mathop{\prod }\limits_{j\mathrm{=1}}^{{N}_{B}}\,{\phi }_{B}({{\bf{r}}}_{j})\mathop{\prod }\limits_{k\mathrm{=1}}^{{N}_{C}}\,{\phi }_{C}({{\bf{r}}}_{k})(({S}_{A}{S}_{B}{)}_{{S}_{AB}}{S}_{C}{)}_{SM}.$$

where the subscript ${\mathfrak{S}}$ denotes a specific set of the good quantum numbers $({S}_{A}{S}_{B}{S}_{C}S)$. When a magnetic field is not applied, the label *M* can be neglected. Accordingly, a candidate of the g.s. can be expanded as

22 $${\Psi }_{{\mathfrak{S}}}=\sum _{{S}_{AB}}\,{d}_{{S}_{AB}}{\psi }_{{\mathfrak{S}},{S}_{AB}},$$

Let $H$ be divided as $H={H}_{{\rm{o}}}+{H}_{{\rm{spin}}}$, where all the spin-dependent interactions are included in ${H}_{{\rm{spin}}}$. Let the indexes $({J}_{-},J,{J}_{+})$ be a cyclic permutation of $(A,B,C)$. Then ${H}_{{\rm{spin}}}={\sum }_{J}{c}_{J2}{\sum }_{1\le i < j\le {N}_{J}}\delta ({{\bf{r}}}_{i}-{{\bf{r}}}_{j}){{\bf{F}}}_{i}^{J}\cdot {{\bf{F}}}_{j}^{J}+$ ${\sum }_{J}{c}_{J{J}_{+}2}{\sum }_{1\le i\le {N}_{J}}{\sum }_{1\le j\le {N}_{{J}_{+}}}\delta ({{\bf{r}}}_{i}-{{\bf{r}}}_{j}){{\bf{F}}}_{i}^{J}\cdot {{\bf{F}}}_{j}^{{J}_{+}}$. When the values of the good quantum numbers in ${\mathfrak{S}}$ are presumed, the coefficients ${d}_{{S}_{AB}}$ can be obtained via a diagonalization of ${H}_{{\rm{spin}}}$ in the space expanded by ${\psi }_{{\mathfrak{S}},{S}_{AB}}$. The matrix elements are

23 $$\begin{array}{rcl}\langle {\psi }_{{\mathfrak{S}},{S}_{AB}\text{'}}|{H}_{{\rm{spin}}}|{\psi }_{{\mathfrak{S}},{S}_{AB}}\rangle  & \equiv  & {H}_{{S}_{AB}\text{'},{S}_{AB}}\\  & = & {\delta }_{{S}_{AB}\text{'}{S}_{AB}}[\sum _{J}\,\frac{1}{2}\int \,{\varphi }_{J}^{4}{\rm{d}}{\bf{r}}{c}_{J2}({T}_{J}-2{N}_{J})+\int \,{\varphi }_{A}^{2}{\varphi }_{B}^{2}{\rm{d}}{\bf{r}}{c}_{AB2}\frac{{T}_{AB}-{T}_{A}-{T}_{B}}{2}]\\  &  & +\,\int \,{\varphi }_{B}^{2}{\varphi }_{C}^{2}{\rm{d}}{\bf{r}}{c}_{BC2}\sum _{{S}_{BC}}\,\bar{w}({S}_{A}{S}_{B}S{S}_{C};{S}_{AB}{S}_{BC})\times \,\bar{w}({S}_{A}{S}_{B}S{S}_{C};{S}_{AB}\text{'}{S}_{BC})\frac{1}{2}({T}_{BC}-{T}_{B}-{T}_{C})\\  &  & +\,\int \,{\varphi }_{C}^{2}{\varphi }_{A}^{2}{\rm{d}}{\bf{r}}{c}_{CA2}\sum _{{S}_{CA}}\,{(-\mathrm{1)}}^{{S}_{AB}\text{'}+{S}_{AB}}\bar{w}({S}_{B}{S}_{A}S{S}_{C};{S}_{AB}{S}_{CA})\\  &  & \bar{w}({S}_{B}{S}_{A}S{S}_{C};{S}_{AB}\text{'}{S}_{CA})\frac{1}{2}({T}_{CA}-{T}_{C}-{T}_{A}),\end{array}$$

where the summation of $J$ covers $A$, $B$ and $C$, $\bar{w}({S}_{A}{S}_{B}S{S}_{C};{S}_{AB}{S}_{BC})=\sqrt{\mathrm{(2}{S}_{AB}+\mathrm{1)(2}{S}_{BC}+\mathrm{1)}}w({S}_{A}{S}_{B}S{S}_{C};{S}_{AB}{S}_{BC})$, the latter is the W-coefficients of Racah, ${T}_{J}={S}_{J}({S}_{J}+\mathrm{1)}$, and so on.

Carrying out the diagonalization of ${H}_{{S}_{AB}\text{'},{S}_{AB}}^{{\mathfrak{S}}}$, the lowest eigenstate is ${\Psi }_{{\mathfrak{S}}}$ and the corresponding energy is denoted as ${E}_{{\mathfrak{S}}}$. Let the presumed values in ${\mathfrak{S}}$ be varied. If ${E}_{{\mathfrak{S}}}$ arrives at its minimum when ${\mathfrak{S}}={{\mathfrak{S}}}_{o}$, then the g.s. ${\Psi }_{gs}={\Psi }_{{{\mathfrak{S}}}_{{\rm{o}}}}$.

To extract information on spin-structure from ${\Psi }_{gs}$, we calculate the averaged angle between the two spins ${S}_{A}$ and ${S}_{B}$ as

24 $$\begin{array}{rcl}{\bar{\theta }}_{AB} & \equiv  & {\cos }^{-1}[\langle {\Psi }_{{\rm{o}}}|{\hat{{\bf{S}}}}_{A}\cdot {\hat{{\bf{S}}}}_{B}|{\Psi }_{{\rm{o}}}\rangle /\sqrt{\langle {\Psi }_{{\rm{o}}}|{\hat{S}}_{A}^{2}|{\Psi }_{{\rm{o}}}\rangle \langle {\Psi }_{{\rm{o}}}|{\hat{S}}_{B}^{2}|{\Psi }_{{\rm{o}}}\rangle }]\\  & = & {\cos }^{-1}[\frac{1}{2\sqrt{{T}_{A}{T}_{B}}}\sum _{{S}_{AB}}\,{d}_{{S}_{AB}}^{2}({T}_{AB}-{T}_{A}-{T}_{B})],\end{array}$$

where ${\hat{{\bf{S}}}}_{J}\equiv {\sum }_{i}\,{{\bf{F}}}_{i}^{J}$ is the operators for the total spin of the *J*-species. Similarly, we have

25 $${\bar{\theta }}_{BC}={\cos }^{-1}\left[\frac{1}{2\sqrt{{T}_{B}{T}_{C}}}\sum _{{S}_{AB},{S}_{AB}\text{'},{S}_{BC}}\,{d}_{{S}_{AB}}{d}_{{S}_{AB}\text{'}}\bar{w}({S}_{A}{S}_{B}S{S}_{C};{S}_{AB}{S}_{BC})\times \,\bar{w}({S}_{A}{S}_{B}S{S}_{C};{S{\prime} }_{AB}{S}_{BC})({T}_{BC}-{T}_{B}-{T}_{C})\right],$$

26 $${\bar{\theta }}_{CA}={\cos }^{-1}\left[\frac{1}{2\sqrt{{T}_{A}{T}_{C}}}\sum _{{S}_{AB},{S}_{AB}\text{'},{S}_{CA}}\,{d}_{{S}_{AB}}{d}_{{S}_{AB}\text{'}}{(-\mathrm{1)}}^{{S}_{AB}\text{'}+{S}_{AB}}\bar{w}({S}_{B}{S}_{A}S{S}_{C};{S}_{AB}{S}_{CA})\times \,\bar{w}({S}_{B}{S}_{A}S{S}_{C};{S}_{AB}\text{'}{S}_{CA})({T}_{CA}-{T}_{C}-{T}_{A})\right].$$

Examples are given below.

### Classical model (Type-I)

Neglecting all spin-independent terms, the spin-dependent energy of the g.s. can be written as

27 $${E}_{{\rm{spin}}}=\langle {\Psi }_{{\rm{o}}}|{H}_{{\rm{spin}}}|{\Psi }_{{\rm{o}}}\rangle =\sum _{J}\,{Q}_{J}\langle \Xi |{\hat{S}}_{J}^{2}-2{N}_{J}|\Xi \rangle +2\sum _{J}\,{Q}_{J{J}_{+}}\langle \Xi |{\hat{{\bf{S}}}}_{J}\cdot {\hat{{\bf{S}}}}_{{J}_{+}}|\Xi \rangle ,$$

where $${Q}_{J}=\int {\varphi }_{J}^{4}{\rm{d}}{\bf{r}}\,{c}_{J2}\mathrm{/2}$$, $${Q}_{J{J}_{+}}=\int {\varphi }_{J}^{2}{\varphi }_{{J}_{+}}^{2}{\rm{d}}{\bf{r}}\,{c}_{J{J}_{+}2}\mathrm{/2}$$.

Based on Eq. (27), we propose a classical model to facilitate qualitative analysis. In this model, the total spin of the $J$-species is considered as a vector ${\overrightarrow{S}}_{J}$ with norm ${S}_{J}$ ranging from 0 to ${N}_{J}$, ${\theta }_{J{J}_{+}}$ is the angle between ${\overrightarrow{S}}_{J}$ and ${\overrightarrow{S}}_{{J}_{+}}$. The magnitudes and orientations of the three ${\overrightarrow{S}}_{J}$ together describe an intuitive picture of the spin-structure. The classical analog of ${E}_{{\rm{spin}}}$ is defined as

28 $${E}_{{\rm{spin}}}^{{\rm{M}}}=\sum _{J}\,{Q}_{J}{S}_{J}^{2}+2\sum _{J}\,{Q}_{J{J}_{+}}{S}_{J}{S}_{{J}_{+}}\,\cos \,{\theta }_{J{J}_{+}},$$

The effect of the inter-species force is embodied by ${Q}_{J{J}_{+}}$. When ${Q}_{J{J}_{+}} < 0$ (attractive), ${\overrightarrow{S}}_{J}$ and ${\overrightarrow{S}}_{{J}_{+}}$ will be lying along the same direction. Whereas when ${Q}_{J{J}_{+}} > 0$ (repulsive), along opposite directions. Note that two of the spins will define a plane and will pull the third lying on the same plane. Therefore, the spin-structures of the 3-species condensates are assumed to be coplanar (this assumption will be checked later). Thus, in what follows, ${\theta }_{AB}+{\theta }_{BC}+{\theta }_{CA}=2\pi$ is given. Accordingly, when $\{{Q}_{J}\}$ and $\{{Q}_{J{J}_{+}}\}$ are given, ${E}_{{\rm{spin}}}^{{\rm{M}}}$ is a function of five variables $({S}_{A},{S}_{B},{S}_{C},{\theta }_{BC},{\theta }_{CA})$. When these variables lead to the minimum of ${E}_{{\rm{spin}}}^{{\rm{M}}}$, they specify a coplanar spin-structure of the g.s. In order to find out the minimum, we calculate the partial derivatives of ${E}_{{\rm{spin}}}^{{\rm{M}}}$. They are given in the appendix.

**Figure 2:**
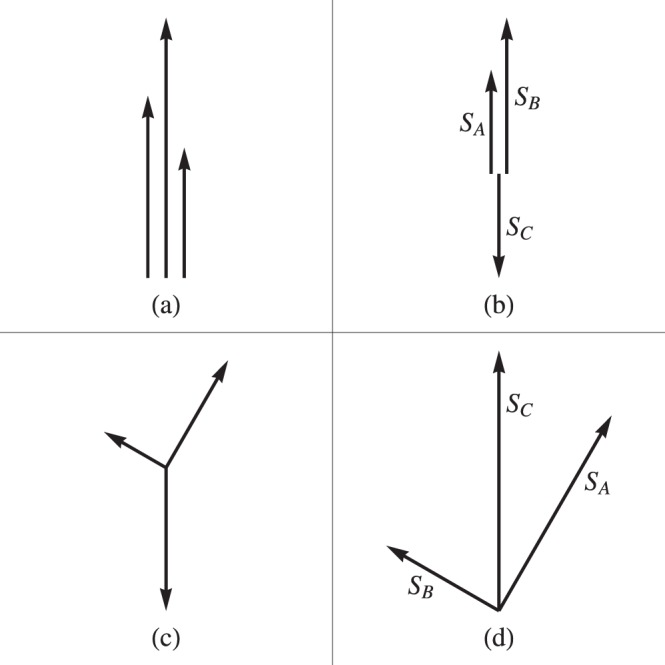
Intuitive pictures of the coplanar spin-structures, where the relative orientations of the spins ${S}_{A}$, ${S}_{B}$ and ${S}_{C}$ are shown.

There are two types of spin-structures. When all $\{{Q}_{J{J}_{+}}\}$ are negative, ${\overrightarrow{S}}_{A}$, ${\overrightarrow{S}}_{B}$ and ${\overrightarrow{S}}_{C}$ would tend to be parallel to each other, i.e., all $\cos \,{\theta }_{J{J}_{+}}=1$ as shown in Fig. 2a. When only one of $\{{Q}_{J{J}_{+}}\}$ is negative, say, ${Q}_{AB}$ is negative, orientations of the spins are shown in Fig. 2b, where $\cos {\theta }_{AB}=1$, $\cos \,{\theta }_{BC}=\,\cos \,{\theta }_{CA}=-\,1$. These two cases belong to the Type-I.

For Type-I

29 $${E}_{{\rm{spin}}}^{{\rm{M}}}=\sum _{J}\,({Q}_{J}{S}_{J}^{2}-2|{Q}_{J{J}_{+}}|{S}_{J}{S}_{{J}_{+}}\mathrm{)}.$$

**Figure 3:**
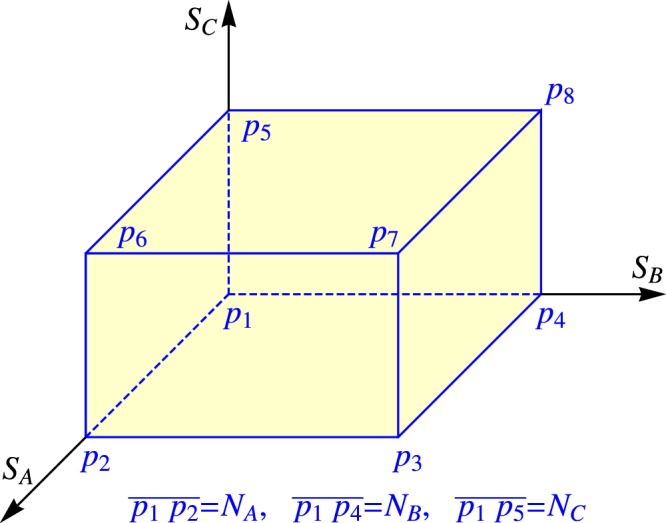
The cuboid formed by the norms of the three spins ${S}_{A}$, ${S}_{B}$, and ${S}_{C}$ each from 0 to ${N}_{J}$.

When ${S}_{J}$ of a species is given at 0, ${N}_{J}$ and in between, let the corresponding phase of the $J$ species be denoted by $p$, $f$ and $q$, respectively. Let $p$ be a point with the coordinates $({S}_{A},{S}_{B},{S}_{C})$ bound by a cuboid as shown in Fig. 3. Let *p*_g.s._ be the point where ${E}_{{\rm{spin}}}^{{\rm{M}}}$ arrives at its minimum. There are the following possibilities.

#### The case *p*_g.s._ is located inside the cuboid (i.e., not on the surfaces of the rectangle)

In this case $0 < {S}_{J} < {N}_{J}$ for all *J*. At the minimum the three equations $\frac{\partial {E}_{{\rm{spin}}}^{{\rm{M}}}}{\partial {S}_{J}}|{p}_{{\rm{g}}{\rm{.s}}.}=0$ are necessary to hold. This leads to a set of homogeneous linear equations for $({S}_{A},{S}_{B},{S}_{C})$ as

30 $${Q}_{J}{S}_{J}-|{Q}_{{J}_{-}J}|{S}_{{J}_{-}}-|{Q}_{J{J}_{+}}|{S}_{{J}_{+}}=\mathrm{0,}\,(J=A,B,C)$$

However, the matrix of this set is in general not singular. Therefore, there is no nonzero solution. Even, for a specific choice of the parameters, the matrix is singular, the nonzero solution can be multiplied by a variable common number $\varsigma$. One can see that ${E}_{{\rm{spin}}}^{{\rm{M}}}$ varies with $\varsigma$ monotonically. In order to minimize ${E}_{{\rm{spin}}}^{{\rm{M}}}$, $\varsigma$ should be given either in its upper or lower limit but not inside. Thus, *p*_g.s._ cannot locate inside the cuboid. *It implies that the three species cannot all be in the q-phase*.

Let a rectangle on the surface of the cuboid be denoted as ${p}_{1}{p}_{4}{p}_{8}{p}_{5}$, etc. (refer to Fig. 3). There are six rectangles classified into two kinds. The three containing the common vertex *P*_1_ belong to the first kind, the other three containing ${p}_{7}$ belong to the second kind.

#### The case *p*_g.s._ is located on a rectangle of the first kind

If this case is realistic, the g.s. would have at least one species in $p$-phase. For instance, if *p*_g.s._ were located on ${p}_{1}{p}_{4}{p}_{8}{p}_{5}$ (i.e., ${S}_{A}=0$, $0\le {S}_{B}\le {N}_{B}$, and $0\le {S}_{C}\le {N}_{C}$), it is necessary to have $\frac{\partial {E}_{{\rm{spin}}}^{{\rm{M}}}}{\partial {S}_{A}}{|}_{{p}_{{\rm{g}}{\rm{.s}}.}}\ge 0$. However, this leads to $-|{Q}_{AB}|{S}_{B}-|{Q}_{CA}|{S}_{C}\ge 0$ which cannot be realized unless ${S}_{B}={S}_{C}=0$. With similar arguments, *p*_g.s._ cannot be located on ${p}_{1}{p}_{5}{p}_{6}{p}_{2}$ and ${p}_{1}{p}_{2}{p}_{3}{p}_{4}$ as well, but it can be located at the point *P*_1_. *It implies that the case with one or two species in P-phase is prohibited, while all species in P is possible*. This fact coincides with the finding found in 2-species condensates, in which the *P*-phase is extremely fragile when it is accompanied by an *f* or a *q*. Therefore, the *P* + *f* or *P* + *q* structures do not exist, but the *P* + *P* structure is allowed^20–23^.

With the prohibition of the above two cases, *p*_g.s._ can only access *P*_1_ and the three rectangles of the second kind, but those edges each being a common edge of two rectangles belonging to two kinds should be excluded.

#### The case *p*_g.s._ = *p*_7_

In this case ${S}_{J}={N}_{J}$ for all $J$ and, accordingly, the structure is denoted as *f*//*f*//*f*. (the symbol //implies that the related spins are either parallel or anti-parallel). The three inequalities $\frac{\partial {E}_{{\rm{spin}}}^{{\rm{M}}}}{\partial {S}_{J}}{|}_{{p}_{7}} < 0$ are required to hold. This leads to the constraints listed at the right of the first row of Table 1. These constraints give the scope of the parameters that supports the *f*//*f*//*f*-structure. The energy of this structure ${E}_{{\rm{spin}}}^{{\rm{M}}}={E}_{fff}^{{\rm{M}}}$ is listed in Table 2. In these tables, we have defined

31 $${\beta }_{J{J}_{+}}\equiv {Q}_{J}{Q}_{{J}_{+}}-{|{Q}_{J{J}_{+}}|}^{2},$$

and

32 $${\beta }_{ABC}\equiv {Q}_{A}{Q}_{B}{Q}_{C}-2|{Q}_{AB}||{Q}_{BC}||{Q}_{CA}|-{Q}_{A}{Q}_{BC}^{2}-{Q}_{B}{Q}_{CA}^{2}-{Q}_{C}{Q}_{AB}^{2}.$$

When all species are ferromagnetic in nature (i.e., all ${Q}_{J} < 0$), the inequality ${N}_{J}{Q}_{J}-{N}_{{J}_{-}}|{Q}_{{J}_{-}J}|-{N}_{{J}_{+}}|{Q}_{J{J}_{+}}| < 0$ holds definitely, and the *f*//*f*//*f* structure is the only choice for the g.s. When some species (say, *J*-species) is polar in nature (i.e., ${Q}_{J} > 0$), the term ${N}_{J}{Q}_{J}$ (representing the intra-interaction) and the other two terms (representing the combined inter-interaction) are competing. Only when $|{Q}_{{J}_{-}J}|$ and $|{Q}_{J{J}_{+}|}$ are sufficiently large the inequality could hold.

**Table 1:** When all ${Q}_{J{J}_{+}} < 0$ or only one ${Q}_{J{J}_{+}} < 0$, the representative possible spin-structures of the g.s. are listed in the first column. The notation *f*//*f*//*q* implies that the *A*, *B* and *C* species are in *f*, *f* and *q*, respectively. The three spins ${S}_{A}$, ${S}_{B}$ and ${S}_{C}$ are either parallel or anti-parallel to each other. The (in)equalities listed in the second column impose a constraint on the parameters so that the associated structure can emerge only in a subspace in the parameter space. In the first row $J=A$, *B* and *C*. $({J}_{-},J,{J}_{+})$ is a cyclic permutation of (*A*, *B*, *C*). The constraints for other possible structures not listed in the table, say, *f*//*q*//*f*, can be obtained by a cyclic permutation of the indexes *A*, *B* and *C*.

Spin-structure	Constraint
*f*//*f*//*f*	${N}_{J}{Q}_{J}-{N}_{{J}_{-}}|{Q}_{{J}_{-}J}|-{N}_{{J}_{+}}|{Q}_{J{J}_{+}}| < 0$
*f*//*f*//*q*	${N}_{A}{Q}_{A}-{N}_{B}|{Q}_{AB}|-{S}_{C}|{Q}_{CA}| < 0$
	$${N}_{B}{Q}_{B}-{S}_{C}|{Q}_{BC}|-{N}_{A}|{Q}_{AB}| < 0$$
	$${S}_{C}{Q}_{C}-({N}_{A}|{Q}_{CA}|+{N}_{B}|{Q}_{BC}|)=0$$
	$${Q}_{C} > 0$$
*f*//*q*//*q*	${N}_{A}{Q}_{A}-{S}_{B}|{Q}_{AB}|-{S}_{C}|{Q}_{CA}| < 0$
	$${S}_{B}={N}_{A}({Q}_{C}|{Q}_{AB}|+|{Q}_{BC}||{Q}_{CA}|)/{\beta }_{BC}$$
	$${S}_{C}={N}_{A}({Q}_{B}|{Q}_{CA}|+|{Q}_{BC}||{Q}_{AB}|)/{\beta }_{BC}$$
	${Q}_{B} > 0$, ${Q}_{C} > 0$, ${\beta }_{BC} > 0$
*p* + *p* + *p*	${\beta }_{ABC}\ge 0,{Q}_{A} > 0$, ${Q}_{B} > 0$, ${Q}_{C} > 0$

**Table 2:** The model energies of the g.s. in various structures.

Model	Energy
${E}_{fff}^{{\rm{M}}}$	${\sum }_{J}\,({Q}_{J}{N}_{J}^{2}-2|{Q}_{J{J}_{+}}|{N}_{J}{N}_{{J}_{+}})$
${E}_{ffq}^{{\rm{M}}}$	$\frac{1}{{Q}_{C}}[{N}_{A}^{2}{\beta }_{CA}+{N}_{B}^{2}{\beta }_{BC}-2{N}_{A}{N}_{B}({Q}_{C}|{Q}_{AB}|+|{Q}_{BC}||{Q}_{CA}|)]$
${E}_{fqq}^{{\rm{M}}}$	$\frac{{N}_{A}^{2}}{{\beta }_{BC}}{\beta }_{ABC}$
${E}_{ppp}^{{\rm{M}}}$	0

#### The case *p*_g.s._ is located in the interior of $\overline{{p}_{7}{p}_{6}}$, $\overline{{p}_{7}{p}_{3}}$ or $\overline{{p}_{7}{p}_{8}}$

When *p*_g.s._ is in the interior of $\overline{{p}_{7}{p}_{3}}$ (the two ends of the edge are not included), ${S}_{A}={N}_{A}$, ${S}_{B}={N}_{B}$, and $0 < {S}_{C} < {N}_{C}$. The associated structure is *f*//*f*//*q*. The two inequalities $\frac{\partial {E}_{{\rm{spin}}}^{{\rm{M}}}}{\partial {S}_{A}}{|}_{{p}_{{\rm{g}}{\rm{.s}}.}} < 0$ and $\frac{\partial {E}_{{\rm{spin}}}^{{\rm{M}}}}{\partial {S}_{B}}{|}_{{p}_{{\rm{g}}{\rm{.s}}.}} < 0$, together with $\frac{\partial {E}_{{\rm{spin}}}^{{\rm{M}}}}{\partial {S}_{C}}{|}_{{p}_{{\rm{g}}{\rm{.s}}.}}=0$ and $\frac{{\partial }^{2}{E}_{{\rm{spin}}}^{{\rm{M}}}}{\partial {S}_{C}^{2}}{|}_{{p}_{{\rm{g}}{\rm{.s}}.}} > 0$ are required. This leads to the constraint listed in the second row of Table 1. This structure can be realized only if ${Q}_{C} > 0$ (i.e., the *C*-species is polar in nature), whereas *Q*_*A*_ and *Q*_*B*_ can be negative or weakly positive. If they are positive and large, the inter-species interaction should be even stronger to ensure that the inequalities hold. The equality for ${S}_{C}$ implies that the intra-force and the inter-force imposed on the *C*-atoms arrive at a balance. The energy ${E}_{ffq}^{{\rm{M}}}$ is given in Table 2. The structures *f*//*q*//*f* (*B*-species in *q*) and *q*//*f*//*f* (*A*-species in *q*) can be similarly discussed. These three together are called the double-*f*-structure (double-*f*-str).

#### The case *p*_g.s._ is located in the interior of the rectangles of the second kind

When *p*_g.s._ is in the interior of ${p}_{7}{p}_{6}{p}_{2}{p}_{3}$, ${S}_{A}={N}_{A}$, $0 < {S}_{B} < {N}_{C}$, and $0 < {S}_{C} < {N}_{C}$. The associated structure is *f*//*q*//*q*. The inequality $\frac{\partial {E}_{{\rm{spin}}}^{{\rm{M}}}}{\partial {S}_{A}}{|}_{{p}_{{\rm{g}}{\rm{.s}}.}} < 0$ together with $\frac{\partial {E}_{{\rm{spin}}}^{{\rm{M}}}}{\partial {S}_{J{\prime} }}{|}_{{p}_{{\rm{g}}{\rm{.s}}.}}=0$ and $\frac{{\partial }^{2}{E}_{{\rm{spin}}}^{{\rm{M}}}}{\partial {S}_{J{\prime} }^{2}}{|}_{{p}_{{\rm{g}}{\rm{.s}}.}} > 0$ ($J{\prime} =B$ and *C*) are required. This leads to the constraint listed in the third row of Table 1. This structure can be realized only if both the $B$- and *C*-species are polar in nature, whereas ${Q}_{A}$ can be negative or weakly positive. Besides, the condition ${Q}_{B}{Q}_{C} > |{Q}_{BC}{|}^{2}$ is necessary. One can prove that the constraint listed in the third row leads to ${\beta }_{ABC} < 0$. Note that ${E}_{ppp}^{{\rm{M}}}=0$ while ${E}_{fqq}^{{\rm{M}}}$ is a product of a positive value and ${\beta }_{ABC}$. Thus, ${\beta }_{ABC} < 0$ is a necessary condition for the *f*//*q*//*q* structure. The structures *q*//*f*//*q* and *q*//*q*//*f* can be similarly discussed. The three together are called the single-*f*-str.

#### The case *p*_g.s._ is located at *p*_1_

When all the three species are polar in nature (${Q}_{A} > 0$, ${Q}_{B} > 0$, ${Q}_{C} > 0$) the first term of ${\beta }_{ABC}$ (i.e., ${Q}_{A}{Q}_{B}{Q}_{C}$) is positive. If the inter-species forces are zero or weak, this positive term would be dominant. This leads to ${\beta }_{ABC}\ge 0$. In this case all the species are in *p* and the structure is therefore denoted as *p* + *p* + *p*. When $\{|{Q}_{J{J}_{+}}|\}$ increases, ${\beta }_{ABC}$ will decrease. Once ${\beta }_{ABC}$ becomes zero, the energy of the single-*f*-str will be lower than ${E}_{ppp}^{{\rm{M}}}$ (refer to Table 2), and the transition *p* + *p* + *p* → single-*f*-str will occur.

With these in mind, the possible spin-strs of the g.s. are *p* + *p* + *p*, single-*f*-str, double-*f*-str, and *f*//*f*//*f* depending on the parameters.

### Spin-structure transition

We aim at the effect caused by the variation of the inter-species forces. Note that ${Q}_{J{J}_{+}}$ can pull the spins of the *J* and *J*_+_ species lying along the same direction (opposite directions) if ${Q}_{J{J}_{+}} < 0$ (>0). Therefore, in general, a stronger $|{Q}_{J{J}_{+}}|$ will cause the appearance of the *f*-phase. Starting from $\{|{Q}_{J{J}_{+}}|\}=0$, the first transition is from *p* + *p* + *p* to a single-*f*-str as mentioned above. Recall that the single-*f*-str must have ${\beta }_{ABC}\le 0$ while the *p* + *p* + *p* has ${\beta }_{ABC} > 0$, therefore ${\beta }_{ABC}=0$ is the critical point of transition. One can prove that the two sets of constraint for two different single-*f*-strs (say, *f*//*q*//*q* and *q*//*f*//*q*) cannot both be satisfied Otherwise, two contradicting inequalities ${\beta }_{ABC} > 0$ and ${\beta }_{ABC}\le 0$ would both hold. This fact implies that, for a given set of parameters, only one of the three single-*f*-strs can survive. Therefore, *p* + *p* + *p* can only transit to a specific single-*f*-str depending on the parameters. Besides, the transitions among the three single-*f*-strs (say, *f*//*q*//$q\to q$//*f*//*q*) are prohibited.

When $\{|{Q}_{J{J}_{+}}|\}$ increase further, a *q*-phase can be changed to a *f*-phase. Therefore, the single-*f*-str → double-*f*-str transition will occur (as shown below). One can prove that the three sets of constraints for the three double-*f*-strs do not compromise with each other as before. Thus, a single-*f*-str can only transit to a specific double-*f*-str depending on the parameters, and the transitions among the three double-*f*-strs are prohibited. When $\{|{Q}_{J{J}_{+}}|\}$ increases further, eventually, the g.s. must be in the *f*//*f*//*f* structure.

With these in mind the increase of $\{|{Q}_{J{J}_{+}}|\}$ will lead to a chain of transitions as *p* + *p* + *p* → single-*f*-str → double-*f*-str → *f*//*f*//*f*.

**Figure 4:**
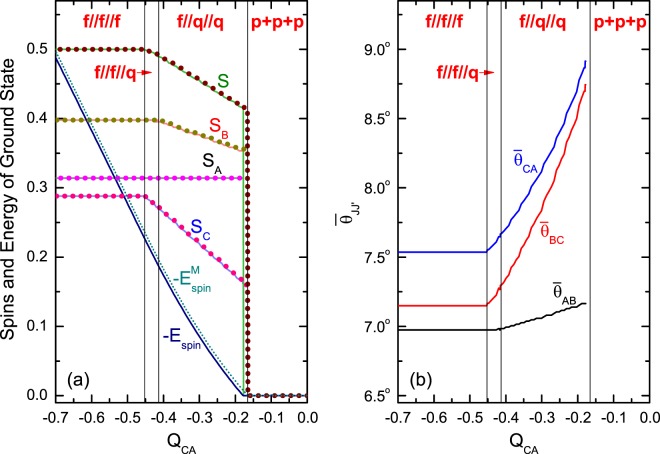
An example for the variation of the spin-structure of Type-I against $${Q}_{CA}$$. The structure is specified by $${S}_{A}/N$$, $${S}_{B}/N$$, $${S}_{C}/N$$, and $$S\mathrm{/(2}N)$$ (where $$N={N}_{A}+{N}_{B}+{N}_{C})$$ in (**a**) and by the angles $${\bar{\theta }}_{AB}$$, $${\bar{\theta }}_{BC}$$ and $${\bar{\theta }}_{CA}$$ (in degree) between them (**b**). The results from the exact diagonalization of $${H}_{{\rm{spin}}}$$ are plotted in solid lines. In (a), the results from the model are plotted in dotted lines, and $${\theta }_{AB}={\theta }_{BC}={\theta }_{CA}=0$$ are assumed. Accordingly, the classical model has $$S={S}_{class}\equiv {S}_{A}+{S}_{B}+{S}_{C}$$ as shown in (**a**). The dimensionless parameters are given as $${N}_{A}=120$$, $${N}_{B}=152$$, $${N}_{C}=110$$, $${Q}_{A}=0.6$$, $${Q}_{B}=0.5$$, $${Q}_{C}=0.77$$, $${Q}_{AB}=-\,0.46$$, $${Q}_{BC}=-\,0.2$$, $${Q}_{CA}$$ is from −0.7 to $$0$$. Since all $$\{{Q}_{J{J}_{+}}\}$$ are given negative, this example represents the case of Fig. 2a.

**Figure 5:**
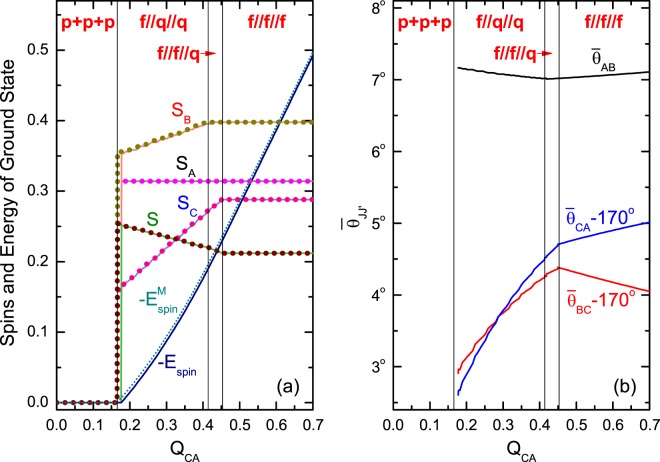
An example similar to Fig. 4 but with ${Q}_{BC}=0.2$ and ${Q}_{CA}$ is from 0 to 0.7. Since only one of $\{{Q}_{J{J}_{+}}\}$ is given negative (*Q*_*AB*_ = −0.46), this example represents the case of Fig. 2b. Accordingly, in the model, ${\theta }_{AB}=0$ and ${\theta }_{BC}={\theta }_{CA}={180}^{\circ }$ are assumed and ${S}_{class}\equiv |{S}_{A}+{S}_{B}-{S}_{C}|$ in (**a**).

Two numerical examples of Type I are shown in Figs. 4 and 5, where the variation of the spin-structure (specified by ${S}_{A}$, ${S}_{B}$, ${S}_{C}$, and ${\bar{\theta }}_{AB}$, ${\bar{\theta }}_{BC}$, ${\bar{\theta }}_{CA}$) against ${Q}_{CA}$ is plotted. The results from the QM calculation are in solid lines, those from the model are in dotted lines. The coincidence is quite well. In particular, the whole chain of transitions predicted via the model are nicely recovered by the QM calculation. The intuitive pictures shown in Fig. 2a,b are also supported by Figs. 4b and 5b. In Fig. 4b the angles are very small <9°, in Fig. 5b the angles are either close to zero or to 180°. Thus, the analysis based on the model is reliable. Note that the model is symmetric with respect to ${Q}_{J{J}_{+}}\leftrightarrow -\,{Q}_{J{J}_{+}}$. This symmetry can be shown by comparing Figs. 4a and 5a.

According to the model, when $|{Q}_{CA}|$ increases, the transition $p$ + $p$ + $p\to f$//$q$//$q$ occurs at $|{Q}_{CA}|={q}_{1}$, where ${E}_{ppp}^{{\rm{M}}}={E}_{fqq}^{{\rm{M}}}$. Thus, ${q}_{1}$ is the solution of the equation

33 $${\beta }_{ABC}=0.$$

In Fig. 4 ${q}_{1}=0.165$ as listed in Table 3. Recall that, for a 2-species BEC, the $p$ + $p\to f$//$q$ transition will occur when ${\beta }_{J{J}_{+}}=0$ ^21–23^. Obviously, Eq. (33) is a generalization of ${\beta }_{J{J}_{+}}=0$. In both equations, the competition of the intra- and inter-interactions is clearly shown.

**Table 3:** The critical values of *Q*_*CA*_ in the chain $p$ + $p$ + $p\to f$//$q$//$q\to f$//$f$//$q\to f$//$f$//$f$. The other parameters are listed in the caption of Fig. 5.

Critical Point	Classical Model	QM Calculation
*q*_1_	0.165	0.176
*q*_2_	0.405	0.409
*q*_3_	0.449	0.446

The transition $f$//$q$//$q\to f$//$f$//$q$ occurs at ${q}_{2}$, where ${E}_{fqq}^{{\rm{M}}}={E}_{ffq}^{{\rm{M}}}$. Thus

34 $${q}_{2}=\frac{1}{{N}_{A}|{Q}_{BC}|}({N}_{B}{Q}_{B}{Q}_{C}-{N}_{B}{|{Q}_{BC}|}^{2}-{N}_{A}{Q}_{C}|{Q}_{AB}|\mathrm{)}.$$

In Fig. 4 ${q}_{2}=0.405$ as listed in Table 3.

The transition $f$//$f$//$q\to f$//$f$//$f$ occurs at ${q}_{3},$ where ${E}_{ffq}^{{\rm{M}}}={E}_{fff}^{{\rm{M}}}$. Thus,

35 $${q}_{3}=\frac{1}{{N}_{A}}({N}_{C}{Q}_{C}-{N}_{B}|{Q}_{BC}|\mathrm{)}.$$

In Fig. 4 ${q}_{3}=0.449$. Recall that, for 2-species BEC with A- and C-atoms, the $f$//$q\to f$//$f$ transition will occur when ${q}_{3}=\frac{1}{{N}_{A}}{N}_{C}{Q}_{C}$ ^21–23^. Thus, the existence of the third species (B-atoms) is helpful to the transition (i.e., the $f$//$f$//$f$ structure can be realized at a smaller $|{Q}_{CA}|$).

The above critical values predicted by the model are close to the values from QM calculation as shown in Table 3 (except ${q}_{1}$, but still acceptable). Thus, the related analytical formulae Eqs. (33, 34, 35) are useful for qualitative evaluation. For other chains of transition, the analytical formulae for the critical points can be similarly obtained.

### Classical model (Type-II)

When all ${Q}_{J{J}_{+}}$ are positive (Fig. 2c) or only one of them is positive (Fig. 2d, where ${Q}_{AB} > 0$), the associated spin-structures are in Type-II. In this type the three spins point at different directions, but they are assumed to be coplanar (${\theta }_{AB}+{\theta }_{BC}+{\theta }_{CA}=2\pi$). The total energy appears as

36 $${E}_{{\rm{spin}}}^{{\rm{M}}}=\sum _{J}\,{Q}_{J}{S}_{J}^{2}+2\sum _{J}\,{Q}_{J{J}_{+}}\text{'}{S}_{J}{S}_{{J}_{+}},$$

where $${Q{\prime} }_{J{J}_{+}}={Q}_{J{J}_{+}}\,\cos \,{\theta }_{J{J}_{+}}$$.

To find out the point *p*_g.s._ where the minimum of ${E}_{{\rm{spin}}}^{{\rm{M}}}$ is located, we first consider the partial derivatives of ${E}_{{\rm{spin}}}^{{\rm{M}}}$ against $\{{S}_{J}\}$ when $\{{Q}_{J}\}$ and $\{{Q{\prime} }_{J{J}_{+}}\}$ are considered as constants. Thus, the situation is the same as for Type-I. With the same arguments as those for Type-I, we deduce that *p*_g.s._ can only access ${p}_{1}$ and the three rectangles of the second kind, but those edges each being a common edge of two rectangles belonging to two kinds should be excluded.

When ${p}_{{\rm{g}}{\rm{.s}}.}={p}_{7}$, every species is fully polarized, but the spins of any two species are in general neither parallel nor antiparallel to each other. Therefore, instead of *f*//*f*//*f*, this type of structure is denoted as *f* + *f* + *f*. The three inequalities $\frac{\partial {E}_{{\rm{spin}}}^{{\rm{M}}}}{\partial {S}_{J}}{|}_{{p}_{{\rm{g}}{\rm{.s}}.}} < 0$ are required which lead to the constraints ${N}_{J}{Q}_{J}+{N}_{{J}_{-}}{Q{\prime} }_{{J}_{-}J}+{N}_{{J}_{+}}{Q{\prime} }_{J{J}_{+}} < 0$, where *J* is for *A*, *B* and *C*. In addition, the two derivatives $\frac{\partial {E}_{{\rm{spin}}}^{{\rm{M}}}}{\partial {\theta }_{BC}}{|}_{{p}_{{\rm{g}}{\rm{.s}}.}}$ and $\frac{\partial {E}_{{\rm{spin}}}^{{\rm{M}}}}{\partial {\theta }_{CA}}{|}_{{p}_{{\rm{g}}{\rm{.s}}.}}$ are required to be zero. These lead to (refer to Eqs. (48) and (49))

37 $$\cos \,{\theta }_{BC}={G}_{BC}({N}_{A}{N}_{B}{N}_{C}),$$

38 $$\cos \,{\theta }_{CA}={G}_{CA}({N}_{A}{N}_{B}{N}_{C}\mathrm{)}.$$

The two angles obtained in this way should ensure that the two second order derivatives given in Eqs. (46) and (47) are positive. When all the ${Q}_{J{J}_{+}} > 0$, from Eqs. (46) and (47) we know that this requirement could be satisfied if ${\theta }_{BC}$ and ${\theta }_{CA}$ are large enough, thereby the repulsion caused by ${Q}_{J{J}_{+}}$ is reduced. Whereas when only one, say, ${Q}_{AB} > 0$ while ${Q}_{BC} < 0$ and ${Q}_{CA} < 0$, ${\theta }_{BC}$ and ${\theta }_{CA}$ should be small enough, thereby the attraction caused by ${Q}_{BC}$ and ${Q}_{CA}$ can be strengthened. Once the angles are known, the three ${Q\text{'}}_{JJ{\prime} }$ are known. Then, the energy ${E}_{{\rm{spin}}}^{{\rm{M}}}={\sum }_{J}\,{Q}_{J}{N}_{J}^{2}+2{\sum }_{J}\,{Q{\prime} }_{J{J}_{+}}{N}_{J}{N}_{{J}_{+}}\equiv {E}_{f+f+f}$ and the subspace of parameters that supports this structure are also known.

When ${p}_{{\rm{g}}{\rm{.s}}.}$ is located in the interior of $\overline{{p}_{7}{p}_{3}}$ as an example, ${S}_{A}={N}_{A}$, ${S}_{B}={N}_{B}$, and the structure is denoted as *f* + *f* + *q*. The constraints appear as (refer to the second row of Table 1):

39 $$\{\begin{array}{l}{N}_{A}{Q}_{A}+{N}_{B}{Q{\prime} }_{AB}+{S}_{C}{Q{\prime} }_{CA} < 0\\ {N}_{B}{Q}_{B}+{S}_{C}{Q{\prime} }_{BC}+{N}_{A}{Q{\prime} }_{AB} < 0\\ {S}_{C}{Q}_{C}+({N}_{A}{Q{\prime} }_{CA}+{N}_{B}{Q{\prime} }_{BC})=0\end{array},$$

The angles are subjected to the two coupled equations (refer to Eqs. (48) and (49))

40 $$\cos \,{\theta }_{BC}={G}_{BC}({N}_{A},{N}_{B},-({N}_{A}{Q{\prime} }_{CA}+{N}_{B}{Q{\prime} }_{BC})/{Q}_{C}),$$

41 $$\cos \,{\theta }_{CA}={G}_{CA}({N}_{A},{N}_{B},-({N}_{A}{Q{\prime} }_{CA}+{N}_{B}{Q{\prime} }_{BC})/{Q}_{C}),$$

where ${Q{\prime} }_{J{J}_{+}}$ depends on the angles. Solving these equations (say, numerically), we can obtain ${\theta }_{BC}$ and ${\theta }_{CA}$. Then, the energy ${E}_{f+f+q}$ and the subspace of parameters that supports this structure can be known as before. The cases of *f* + *q* + *f* and *q* + *f* + *f* can be similarly discussed.

When ${p}_{{\rm{g}}{\rm{.s}}.}$ is located in the interiors of the rectangles of the second kind, say, ${p}_{7}{p}_{6}{p}_{2}{p}_{3}$, then ${S}_{A}={N}_{A}$ and the structure is denoted as *f* + *q* + *q*. The constraint imposed on this structure is listed in the third row of Table 1 but with $-|{Q}_{J{J}_{+}}|$ being replaced by ${Q}_{JJ}\text{'}$. In addition, the two coupled equations

42 $$\cos \,{\theta }_{BC}={G}_{BC}({N}_{A}{S}_{B}{S}_{C}),$$

43 $$\cos \,{\theta }_{CA}={G}_{CA}({N}_{A}{S}_{B}{S}_{C}),$$

are required to be satisfied. Then ${S}_{B}$, ${S}_{C}$, together with the angles can be known, thereby ${E}_{f+q+q}^{{\rm{M}}}$ is known.

When all ${Q}_{J} > 0$, if the strengths of the inter-species interaction are weak, all the three ${E}_{f+q+q}^{{\rm{M}}}$, ${E}_{q+f+q}^{{\rm{M}}}$, and ${E}_{q+q+f}^{{\rm{M}}}$ will be larger than zero, in this case ${p}_{{\rm{g}}{\rm{.s}}.}={p}_{1}$ and the structure is *p* + *p* + *p*.

A comparison of the results from the model and from the diagonalization of ${H}_{{\rm{spin}}}$ is shown in Table 4.

**Table 4:** For the structure *f* + *f* + *f* of the Type-II., the angles (in degrees) between the spins against the increase of *Q*_*CA*_. The data for ${\theta }_{J{J}_{+}}$ are from the model (refer to Eqs. (37) and (38)), those for ${\bar{\theta }}_{J{J}_{+}}$ are from the diagonalization of ${H}_{{\rm{spin}}}$ (refer to Eqs. (24), (25) and (26)). The parameters are given as ${N}_{A}=120$, ${N}_{B}=152$, ${N}_{C}=110$, *Q*_*A*_ = −0.6, *Q*_*B*_ = −0.5, *Q*_*C*_ = −0.77, *Q*_*AB*_ = 0.3, *Q*_*BC*_ = 0.4, *Q*_*CA*_ is from 0.3 to 0.8.

*Q*_*CA*_	*θ*_*CA*_	${\bar{{\boldsymbol{\theta }}}}_{{\boldsymbol{CA}}}$	*θ*_*BC*_	${\bar{{\boldsymbol{\theta }}}}_{{\boldsymbol{BC}}}$	*θ*_*AB*_	${\bar{{\boldsymbol{\theta }}}}_{{\boldsymbol{AB}}}$	${\bar{{\boldsymbol{\theta }}}}_{{\boldsymbol{CA}}}+{\bar{{\boldsymbol{\theta }}}}_{{\boldsymbol{BC}}}+{\bar{{\boldsymbol{\theta }}}}_{{\boldsymbol{AB}}}$
0.3	81.6	$81.8$	$144.2$	$144.0$	$134.3$	$134.1$	$359.9$
0.4	$111.3$	$111.1$	$132.7$	$132.5$	$116.0$	$116.4$	$360.0$
0.5	$126.9$	$126.7$	$127.9$	$127.6$	$105.3$	$105.5$	$359.8$
0.6	$136.8$	$136.7$	$125.8$	$125.5$	$97.5$	$97.6$	$359.8$
0.7	$143.7$	$143.5$	$125.1$	$124.7$	$91.2$	$91.6$	$359.8$
0.8	$148.9$	$148.5$	$125.3$	$124.6$	$85.8$	$86.6$	$359.7$

Table 4 demonstrates that the results given by Eqs. (24), (25) and (26) are quite accurate. In particular, the sum of the three $\{{\bar{\theta }}_{J{J}_{+}}\}$ given in the last column is very close to 2*π*. This supports the assumption of coplanar structure.

### Final remarks

Features of the spin-structures of 3-species condensates with spin-1 atoms have been extracted from a model and have been checked via a QM calculation. Note that the effect of the spatial wave function is embodied in the factors $\int \,{\varphi }_{J}^{4}{\rm{d}}{\bf{r}}$ and $\int \,{\varphi }_{J}^{2}{\varphi }_{{J}_{+}}^{2}{\rm{d}}{\bf{r}}$ included in ${Q}_{J}$ and ${Q}_{JJ+}$, respectively. Since we do not aim at specific kinds of atoms, they are just considered as parameters to avoid the solving of the CGP (of course, this step is necessary when specific species are aimed). The results from the model are found to be consistent with those from the QM calculation. In summary:

- The structures can be described by the norms of the three spins $\{{S}_{J}\}$ and the average angles $\{{\bar{\theta }}_{J{J}_{+}}\}$ between them. When the three species are polar in nature (i.e., all ${c}_{J2} > 0$) and the inter-forces are weak, the mixture is in the $p$ + $p$ + $p$ phase.
- The spin-structures not in $p$ + $p$ + $p$ are all coplanar. They can be first classified according to the relative orientations of $\{{S}_{J}\}$ as intuitively shown in Fig. 2. The case that all inter-forces are attractive (i.e., all ${c}_{JJ{\prime} 2} < 0$) is shown in Fig. 2a, only one is attractive (say, ${c}_{AB2} < 0$,) in Fig. 2b, all are repulsive (all ${c}_{JJ{\prime} 2} > 0$) in Fig. 2c, and only one is repulsive (say, ${c}_{AB2} > 0$) in Fig. 2d.
- The spin-structures can be further classified according to the norms of the spin. In addition to $p$ + $p$ + $p$, there are other three structures, namely, the single-$f$-str (where one species is in $f$), the double-$f$-str (two species in $f$), and the $f$ + $f$ + $f$ (all in $f$). Note that the single-$p$-str, the double-$p$-str, and the $q$ + $q$ + $q$ do not exist. Thus, $p$ and $f$ (or $p$ and $q$) cannot coexist, just as found in 2-species BEC. If not in $p$ + $p$ + $p$, at least a species must be fully polarized, also similar to 2-species cases.
- Starting from the $p$ + $p$ + $p$, when $|{c}_{JJ{\prime} 2}|$ increases, more species will tend to be in $f$-phase. Therefore, a chain of phase-transitions $p$+$p$ + $p\to f$ + $q$ + $q\to f$ + $f$ + $q\to f$+$f$ + $f$ will occur. In the parameter space, there are a number of critical surfaces. When the point (representing a set of parameters) vary and pass through one of the surfaces, a transition will occur. For Type-I (Fig. 2a,b) the equations describing the surfaces have been quite accurately obtained (refer to Eqs. (33), (34) and (35)). Thus, the critical points at which the transitions occur can be predicted. Moreover, the analytical formulae demonstrate the competition among contradicting physical factors, thereby the inherent physics could be understood better. For Type-II (Fig. 2c,d), analytical analysis based on the model becomes complicated. Nonetheless, the results from the model have been checked to be also valid.
- The spin-structures found above might also appear in *K*-species BEC ($K > 3$). For examples, the case of Fig. 2a might appear if all inter-species interactions are attractive. Figure 2b might appear if the species are divided into two groups and the inter-species interactions inside each group are attractive while those between the two groups are repulsive. When the species are divided into three groups ${G}_{A}$, ${G}_{B}$ and ${G}_{C}$, and all the inter-species interactions inside each group are attractive, and (i) if all the inter-species interactions between any pair of groups are repulsive, then Fig. 2c might appear. (ii) if those between ${G}_{B}$ and ${G}_{C}$, and between ${G}_{C}$ and ${G}_{A}$ are attractive, but those between ${G}_{A}$ and ${G}_{B}$ are repulsive, then Fig. 2d might appear. Of course, in addition to those plotted in Fig. 2a to 2d, more complicated structures might exist (say, non-coplanar structures).
- Recall that, for 2-species BEC, the phases $p$ + $f$ and $p$ + $q$ are prohibited. This originates from the fragility of the $p$-phase when it is accompanied with a $f$- or a $q$-phase. The fragility is recovered in 3-species BEC (say, *p* + *f* + *q* is prohibited) and is believed to hold also for $K > 3$ cases. It implies that any species of the mixture cannot be in $p$-phase, except that all species are in $p$-phase. Furthermore, for 2-species (3-species), the phase $q$ + $q$ ($q$ + $q$ + $q$) is prohibited. The latter implies that the point with the coordinates $\{{S}_{J}\}$ is located in the interior of a cuboid. In this case the requirement $\{\frac{\partial {E}_{{\rm{spin}}}^{{\rm{M}}}}{\partial {S}_{J}}=0\}$ would lead to the fact that $\{{S}_{J}\}$ should obey a set of homogeneous linear equations (refer to Eqs. (51) and (30)). This leads to the prohibition of the interior. This prohibition is believed to also hold for $K\mathrm{ > 3}$ cases (i.e., the *K*-species cannot all be in $q$-phase). Nonetheless, the predictions on *K*-species BEC remain to be checked.

## Appendix

From the spin-dependent energy of the model given in Eq. (28) we have the derivatives:

44 $$\frac{\partial {E}_{{\rm{spin}}}^{{\rm{M}}}}{\partial {\theta }_{BC}}=-\,2[{S}_{A}{S}_{B}{Q}_{AB}\,\sin ({\theta }_{BC}+{\theta }_{CA})+{S}_{B}{S}_{C}{Q}_{BC}\,\sin \,{\theta }_{BC}],$$

45 $$\frac{\partial {E}_{{\rm{spin}}}^{{\rm{M}}}}{\partial {\theta }_{CA}}=-\,\mathrm{2[}{S}_{A}{S}_{B}{Q}_{AB}\,\sin ({\theta }_{BC}+{\theta }_{CA})+{S}_{C}{S}_{A}{Q}_{CA}\,\sin \,{\theta }_{CA}],$$

46 $$\frac{{\partial }^{2}{E}_{{\rm{spin}}}^{{\rm{M}}}}{\partial {\theta }_{BC}^{2}}=-\,\mathrm{2[}{S}_{A}{S}_{B}{Q}_{AB}\,\cos ({\theta }_{BC}+{\theta }_{CA})+{S}_{B}{S}_{C}{Q}_{BC}\,\cos \,{\theta }_{BC}],$$

47 $$\frac{{\partial }^{2}{E}_{{\rm{spin}}}^{{\rm{M}}}}{\partial {\theta }_{CA}^{2}}=-\,\mathrm{2[}{S}_{A}{S}_{B}{Q}_{AB}\,\cos ({\theta }_{BC}+{\theta }_{CA})+{S}_{C}{S}_{A}{Q}_{CA}\,\cos \,{\theta }_{CA}\mathrm{]}.$$

Note that the coupled equations $\frac{\partial {E}_{{\rm{spin}}}^{{\rm{M}}}}{\partial {\theta }_{BC}}=0$ and $\frac{\partial {E}_{{\rm{spin}}}^{{\rm{M}}}}{\partial {\theta }_{CA}}=0$ have a trivial solution: both ${\theta }_{BC}$ and ${\theta }_{CA}$ are equal to 0 or *π*, and a non-trivial solution as

48 $$\cos \,{\theta }_{BC}={G}_{BC}({S}_{A}{S}_{B}{S}_{C})\equiv \frac{{({S}_{A}{Q}_{AB}{Q}_{CA})}^{2}-{({S}_{B}{Q}_{AB}{Q}_{BC})}^{2}-{({S}_{C}{Q}_{BC}{Q}_{CA})}^{2}}{2{S}_{B}{S}_{C}{Q}_{AB}{Q}_{BC}^{2}{Q}_{CA}},$$

49 $$\cos \,{\theta }_{CA}={G}_{CA}({S}_{A}{S}_{B}{S}_{C})\equiv \frac{-{({S}_{A}{Q}_{AB}{Q}_{CA})}^{2}+{({S}_{B}{Q}_{AB}{Q}_{BC})}^{2}-{({S}_{C}{Q}_{BC}{Q}_{CA})}^{2}}{2{S}_{A}{S}_{C}{Q}_{AB}{Q}_{BC}{Q}_{CA}^{2}}.$$

Besides, one can prove the following useful relation

50 $$\sin \,{\theta }_{CA}=\frac{{S}_{B}{Q}_{BC}}{{S}_{A}{Q}_{CA}}\,\sin \,{\theta }_{BC}.$$

We further have

51 $$\frac{\partial {E}_{{\rm{spin}}}^{{\rm{M}}}}{\partial {S}_{J}}=\mathrm{2[}{Q}_{J}{S}_{J}+{Q}_{{J}_{-}J}\,\cos \,{\theta }_{{J}_{-}J}{S}_{{J}_{-}}+{Q}_{J{J}_{+}}\,\cos \,{\theta }_{J{J}_{+}}{S}_{{J}_{+}}],\,(J=A,B,C)$$

52 $$\frac{{\partial }^{2}{E}_{{\rm{spin}}}^{{\rm{M}}}}{\partial {S}_{J}^{2}}=2{Q}_{J}.$$

The above partial derivatives of ${E}_{{\rm{spin}}}^{{\rm{M}}}$ are essential in the search of the g.s.
